# Effects of Pulsed Radiofrequency Source on Cardiac Ablation

**DOI:** 10.3390/bioengineering10020227

**Published:** 2023-02-08

**Authors:** Marcello Iasiello, Assunta Andreozzi, Nicola Bianco, Kambiz Vafai

**Affiliations:** 1 Dipartimento di Ingegneria Industriale, Università degli Studi di Napoli Federico II, Piazzale Tecchio 80, 80125 Napoli, Italy; 2 Department of Mechanical Engineering, University of California, 900 University Avenue, Riverside, CA 92521, USA

**Keywords:** porous media, bioheat, RF catheter ablation, pulsating heat

## Abstract

Heart arrhythmia is caused by abnormal electrical conduction through the myocardium, which in some cases, can be treated with heat. One of the challenges is to reduce temperature peaks—by still guaranteeing an efficient treatment where desired—to avoid any healthy tissue damage or any electrical issues within the device employed. A solution might be employing pulsed heat, in which thermal dose is given to the tissue with a variation in time. In this work, pulsed heat is used to modulate induced temperature fields during radiofrequency cardiac ablation. A three-dimensional model of the myocardium, catheter and blood flow is developed. Porous media, heat conduction and Navier–Stokes equations are, respectively, employed for each of the investigated domains. For the electric field, solved via Laplace equation, it is assumed that the electrode is at a fixed voltage. Pulsed heating effects are considered with a cosine time-variable pulsed function for the fixed voltage by constraining the product between this variable and time. Different dimensionless frequencies are considered and applied for different blood flow velocity and sustained voltages. Results are presented for different pulsed conditions to establish if a reasonable ablation zone, known from the obtained temperature profiles, can be obtained without any undesired temperature peaks.

## 1. Introduction

Cardiovascular diseases are known to be the major cause of death currently. One of these is caused by arrhythmias, that is caused by an abnormal electric conduction through the heart, causing an anomalous heartbeat. Consequently, some complications might arise, for instance, heart failure or strokes. This disease can be treated by means of various techniques. One of these techniques is based on the usage of heat induced by radiofrequencies (RF) to destroy the abnormal conduction pathways through the myocardium. This technique is also widely used for the treatment of other diseases like cancer [1] and it is briefly described in the following with references to cardiac ablation. Starting from an electrophysiological analysis of the heartbeat, a needle within a catheter is percutaneously introduced via a femoral vein. This needle is located within particular parts of the cardiac ablation where some abnormal conduction pathways arise; therefore, a RF current is induced via the needle in order to perform myocardium tissue ablation to avoid abnormal conduction.

In recent years, many efforts have been done to improve the currently used models and procedures for RF ablation. For example, a spherical electrode surrounded by the tissue has been proposed by Haines and Watson [2]. By employing a similar model, blood flow circulation has been included by Labonté et al. [3]. The effect of blood circulation by considering blood as a part of the domain has been considered on other papers [4,5,6]. Since this process can influence the electric and temperature fields, another model that also includes the ablation catheter with a thermistor on the tip has been proposed by Lai et al. [7]. In particular, they analyzed different target temperatures, in order to obtain a relationship between lesion dimensions and ablation time for various cases. The effect of the blood flow as a heat sink is also important; as remarked in Gonzàlez-Suàrez and Berjano [8]. They compared different models, showing that avoiding blood flow modeling with mass, momentum and energy equations might cause discrepancies with experimental results. Pulsatile blood flow effects have been studied in [9], where it has been shown that these effects are not relevant. Tissue deformation during RF cardiac ablation modeling has been analyzed in [10] by considering such effect due to the heartbeat that might cause some electrode displacement. Finally, a comprehensive model based on the porous media theory has been proposed by Iasiello et al. [11]. The myocardium was treated as a perfused porous media, and they invoked the Local Thermal Non-Equilibrium (LTNE) assumption for the two-phase myocardium.

One of the challenges related to RF cardiac ablation is to avoid temperature peaks. This is because healthy cells might be destroyed, or there might be undesired effects like coagulation on the electrode, steam popping, perforation and so on [12]. A solution could be to modulate the RF source and then the applied heat, in order to avoid temperature peaks while the same results in terms of ablation zone are achieved. Modulating heat has been proposed in other hyperthermia applications. For spinal pain, Sluijter [13,14] showed that modulated heat does not cause neural damage since lower temperatures are obtained. Microwave ablation pulsating protocols have also been proposed in the literature. Bedoya et al. [15] performed experiments on both ex vivo bovine and in vivo porcine livers, concluding that a pulsed protocol can create larger ablation zones even when less average power is delivered. For RF tumor ablation, various studies have been presented through the years. Goldberg et al. [16] performed both in vivo and ex vivo experiments with variable peak current duration and fixed duration time and vice versa, obtaining larger tumor ablation zones. Fukushima et al. [17] treated hepatocellular carcinoma with either temperature control or impedance control variable through time, showing that impedance control procedure makes larger ablation zones and reduced ablation times. Different waveforms for pulsating protocols have been numerically analyzed by Zhang et al. [18], showing that the half-square wave provides the larger ablation area. Andreozzi et al. [19], with references to a generic heat source for hyperthermia treatment, compared different time-variable applied heat sources by assuming equal delivered energy. It has been shown that moduling the heat would avoid temperature peaks by obtaining similar ablation areas. A comparison between bioheat models to predict RF ablation under pulsating heat condition has been done in [20] at constrained energy. For microwave tumor ablation, Radosevic et al. [21] compared coagulation sizes obtained from pulsed and continuous models. By assuming equal total energy, different on/off duty cycles by considering various time combinations have been applied. Their numerical outcomes have been compared with experimental in vivo and ex vivo results, too. In their work, it is demonstrated that, even if predictions are in according with experiments, there is no improved coagulation zone or sphericity with the pulsed protocols employed there.

Recently, some studies showed the importance of pulsating protocols for RF cardiac ablation [10,22]. Different pulses durations, say 20 s and 30 s, for intervals from 5 to 70 s, have been analyzed in [22], showing that a second RF pulse would increase the lesion depth, even if temperature peaks are higher. Similar conclusions have been found in [10] by accounting for tissue deformation too. As per the authors’ knowledge, for RF cardiac ablation, no study has analyzed different time-variable protocols by assuming that these vary with continuous functions like sine-wave functions. Therefore, in this study, the use of modulating heat in terms of applied thermal dose variation with time to improve the ablation zone for cardiac RF ablation is proposed. Such modulating heat effects on RF cardiac ablation are analyzed. The model takes into account myocardium tissue, free blood flow and the catheter used for ablation. Governing equations are written for all the analyzed domains, with porous media equations applied within the myocardium region. The effects of modulating heat are analyzed by employing a pulsed function under different frequencies, and also effects of the blood flow velocity and applied voltage are presented.

## 2. Methodology

### 2.1. Geometry and Governing Equations

**Figure 1:**
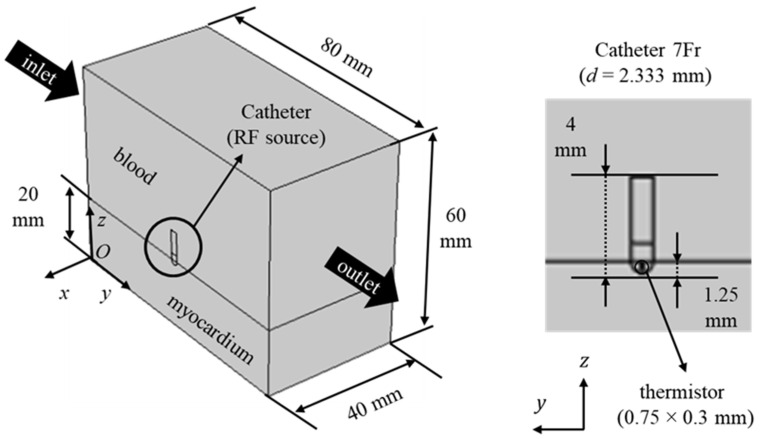
Computational domain for RF cardiac ablation.

The computational domain employed here is shown in Figure 1, together with dimensions taken from [8,11]. In particular, cardiac tissue thickness and blood flow domain dimensions are chosen from the literature [8,23] after the authors performed a sensitivity analysis in order to neglect edge effects. The catheter is embedded in the cardiac tissue with a certain depth, that is 1.25 mm [24]. This value has been taken from Schutt et al. [24], where the authors analyzed different insertion depth that range between 0.75 and 2.50 mm. In particular, the catheter has been divided into the thermistor, the electrode and the catheter itself, because catheters present a temperature distribution due to the different thermophysical properties of the parts that would cause this distribution [25]. Even if thermistor is referred to temperature-controlled situations, we have included this in our model since a thermistor would be useful to switch from temperature-controlled to voltage-controlled procedures, and also to monitor temperature during the process. Thermistor dimensions are taken from Schutt et al. [24]. As shown in [25], the thermistor presents a more or less uniform temperature that does not present any significant effects on temperature fields. The catheter has been chosen to be equal to 7Fr (French Scale) as in [8]; this corresponds to 2.333 mm since one French scale is equal to three times the diameter in millimeters. Governing equations are the same as those given in Iasiello et al. [11]. Due to the presence of the electric field, the electromagnetic wave propagation equation should be solved. However, for radiofrequencies, the DC approach can be used since waves propagate near a small area in the proximity of the electrode [26,27,28]. By neglecting displacement currents and resistive tissues, one can write the voltage equation as follows:

(1) $$\nabla \cdot \left(\sigma \nabla V\right)=0$$

As such, it is possible to derive the heat generation source term in W/m^3^, ${\dot{Q}}_{RF}=\sigma {\left|E\right|}^{2}/2$. For the catheter region, the transient-state heat conduction equation is solved:

(2) $$\rho C\frac{\partial T_{c}}{\partial t}=k\nabla^{2}T_{c}$$

For the blood flow region, under the assumption of Newtonian laminar flow [29] with no natural convection and radiation effects, mass, momentum and energy equations are:

(3) $$\frac{\partial \rho }{\partial t}+\nabla \cdot \left(\rho u\right)=0$$

(4) $$\rho \left(\frac{\partial u}{\partial t}+u\cdot \nabla u\right)=-\nabla p+\mu \nabla^{2}u$$

(5) $$\rho C\left(\frac{\partial T_{b}}{\partial t}+u\cdot \nabla T_{b}\right)=\nabla \cdot \left(k\nabla T_{b}\right)$$

For the myocardium, the porous media approach is used [11,30,31,32]. This approach has been widely used for several biological issues [33,34,35,36]. Even if many models, starting from Pennes’ bioheat equation in 1949, have been proposed through the years [33,36], the LTNE porous media approach is here employed because compared to models like Pennes’ bioheat equation it has a better view of the local microstructure effect, even if it is more complex to implement and requires more coefficients to close governing equations than other models. This model has been widely used in other engineering applications [37,38,39,40] and also applied to bioheat transfer problems. The myocardium is assumed to be made of a fluid phase, that is the blood that passes through the muscle, and a solid phase, that is made up by tissue cells and so on. Governing equations are written for a Representative Elementary Volume (REV) [41] by reasonably assuming that there are no microscopic inertial flow effects, no natural convection and no radiation, and by making references to the Local Thermal Non-Equilibrium (LTNE) assumption between the two phases [42,43].

(6) $$\frac{\partial \rho }{\partial t}+\nabla \cdot \rho \langle u\rangle =0$$

(7) $$\frac{\rho }{\varepsilon }\left(\frac{\partial \langle u\rangle }{\partial t}+\frac{\langle u\rangle }{\varepsilon }\cdot \nabla \langle u\rangle \right)=-\nabla \langle p\rangle +\frac{\mu }{\varepsilon }\nabla^{2}\langle u\rangle -\frac{\mu }{K}\nabla \langle u\rangle$$

(8) $$\varepsilon {\left(\rho c\right)}_{eff}\left(\frac{\partial {\langle T\rangle }_{b}}{\partial t}+u\cdot \nabla {\langle T\rangle }_{b}\right)=\nabla \cdot \left(\varepsilon k\nabla {\langle T\rangle }_{b}\right)+h_{c}S_{v}\left({\langle T\rangle }_{b}-{\langle T\rangle }_{t}\right)+\varepsilon \frac{\sigma {\left|E\right|}^{2}}{2}$$

(9) $$\left(1-\varepsilon \right){\left(\rho c\right)}_{eff}\frac{\partial {\langle T\rangle }_{t}}{\partial t}=\nabla \cdot \left[\left(1-\varepsilon \right)k\nabla {\langle T\rangle }_{t}\right]-h_{c}S_{v}\left({\langle T\rangle }_{b}-{\langle T\rangle }_{t}\right)+\left(1-\varepsilon \right)\frac{\sigma {\left|E\right|}^{2}}{2}$$

where the symbol < > stands for the volumetric average form of a generic variable and the term *σ*|**E**|^2^/2 represents the heat source due to Joule heating obtained from Equation (1). In these equations, effective properties are considered as volumetric weighted average through the domain. The effects of metabolism are here neglected [44]. In these applications, it might happen that temperatures go above 100 °C, so, for the sake of completeness, features referred to what happens during evaporation are reported here when the model is introduced. However, from preliminary calculations, it has been shown that temperatures are not higher than 100 °C for the cases here investigated, so any effects like inter-tissue boiling that might arise around 100 °C [45,46,47], are here neglected.

### 2.2. Boundary Conditions and Thermophysical Properties

Boundary conditions employed are the same as those used by Iasiello et al. [11]. Initially, it is assumed that the entire system is at *T* = *T*_0_, with *V* = 0 V. After *t* > 0, for the blood flow region, it is assumed that the flow enters as a plug flow with a velocity equal to |**u**|_in_, then exits with an outlet pressure of 0 mmHg. It is emphasized that no relevant effects from blood pulsatility has been found in [9], so the plug flow assumption is reliable. At the boundaries of the blood flow region, slip condition is used to replace the symmetry conditions. For the energy equation, a uniform inlet temperature *T_in_* of 37 °C is assumed, while continuity is applied at the free flow/porous media interface. In the porous media region, extremities are assumed to be at *T*_0_ since they are sufficiently far from the thermal disturbance in the catheter. For the voltage field, a uniform voltage *V_in_* is assumed at the catheter part embedded into the myocardium [8], while a dispersive electrode condition of 0 V is applied elsewhere. Finally, thermophysical properties are taken from the literature and presented in Table 1. These are taken after González-Suárez and Berjano [8] and Iasiello et al. [11]. In particular, electric conductivity, thermal conductivity and volumetric heat capacity variation with temperature are taken into account as follows:

(10) $$\sigma \left({\langle T\rangle }_{t}\right)=\left\{\begin{array}{l} 0.541e^{0.015\left({\langle T\rangle }_{t}-T_{0}\right)}\\ 1.371-0.274\left({\langle T\rangle }_{t}-T_{ev}\right)\\ 1.371\cdot 10^{-4} \end{array}\right.\begin{array}{c} 0\;{}^{\circ}C<{\langle T\rangle }_{t}\leq 100\;{}^{\circ}C\\ 100\;{}^{\circ}C<{\langle T\rangle }_{t}\leq 105\;{}^{\circ}C\\ {\langle T\rangle }_{t}>105\;{}^{\circ}C \end{array}$$

(11) $$k\left({\langle T\rangle }_{t}\right)=\left\{\begin{array}{l} 0.531+0.0012\left({\langle T\rangle }_{t}-T_{0}\right)\\ 0.606 \end{array}\right.\begin{array}{c} 0\;{}^{\circ}C<{\langle T\rangle }_{t}\leq 100\;{}^{\circ}C\\ {\langle T\rangle }_{t}>100\;{}^{\circ}C \end{array}$$

where *λ* = 2257 kJ/kg, *c_H2O_* = 0.75 and (*ρc*)*_l_* = 3.298 MJ/m^3^ K and (*ρc*)*_g_* = 0.798 MJ/m^3^ K. Reference temperature *T*_0_ is equal to 37 °C, while *T_ev_* is the water evaporation temperature of 100 °C. For the volumetric heat capacity, an equivalent method is used to consider vaporization effects on the heat capacity for both phases (Equation (12)) by assuming that ∆*T* = 1 °C. Porous media properties are taken from Iasiello et al. [11]. In particular, the permeability values for the tissues are taken from references [30,33,48]. Interfacial heat transfer coefficient and specific surface area are taken from Yuan [49] by assuming that capillary diameters in the myocardium are 8 µm ρ

(12) $${\left(\rho c\right)}_{eff}\left({\langle T\rangle }_{t,b}\right)=\left\{\begin{array}{l} {\left(\rho c\right)}_{l}\\ \frac{\rho_{b}\lambda c_{H_{2}O}}{\Delta T}\\ {\left(\rho c\right)}_{g} \end{array}\right.\begin{array}{c} 0\;{}^{\circ}C<{\langle T\rangle }_{t,b}\leq 99\;{}^{\circ}C\\ 99\;{}^{\circ}C<{\langle T\rangle }_{t,b}\leq 100\;{}^{\circ}C\\ {\langle T\rangle }_{t,b}>100\;{}^{\circ}C \end{array}$$

### 2.3. Modulated Heat Transfer Modeling

**Figure 2:**
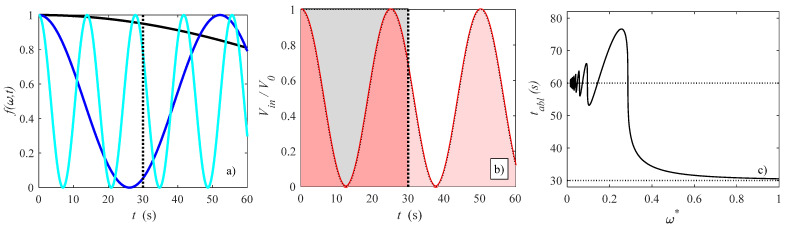
(**a**) different angular frequency functions, where black, blue and light blue lines stand for ω* = 1.000, 0.125, and 0.033, respectively, (**b**) input-to-uniform voltage ratio for ω* = 0.250, where the regions in black and red stand for uniform and pulsed, respectively, and (**c**) solution of Equation (15); the dotted line in (**a**) refers to *t_abl_*(ω* = 1).

Modulated heat transfer effects are considered by temporal variation of the voltage. In particular, a pulsed function that follows a cosine law is employed. Taking *V*_0_ as the applied voltage without angular frequency (that corresponds to *V_in_* for the steady-applied voltage case), then the applied voltage for the time-variable case becomes equal to

(13) $$V_{in}=V_{0}f\left(\omega ,t\right)=V_{0}\left[\frac{1}{2}+\frac{1}{2}\cos\left(\omega t\right)\right]$$

with the applied voltage *V_in_ = V*_0_ *f*(*ω,t*). The amplitude of this function is *V*_0_, while *f*(*ω,t*) presents a unitary amplitude. The reason why this kind of cosine function is used is because this function varies between 0 and 1, and it is equal to 1 for *t* = 0 that corresponds to the beginning of the transient procedure. In order to compare various pulsed scenarios, a dimensionless angular frequency that varies between 0 and 1 is first defined, while a reference angular frequency *ω*_0_ needs to be defined. This reference angular frequency refers to a quasi-steady case because if we use a steady case, then the angular frequency *ω* would have to approach 0. The reference angular frequency definition is reported in the following. We assume that for the steady case the ablation time *t_abl_* is equal to *t_abl_* = 30 s. For the quasi-steady case, we assume *f*(*ω*,*t_abl_* = 30) = 0.95 (see Equation (13)), thus a 5% deviation is assumed in order to avoid *ω*_0_→0. With these assumptions, if we solve the cosine function in Equation (13) we obtain that *ω*_0_ = 0.015 rad/s that is the reference quasi-steady pulsed function. This value roughly corresponds to 2.4·10^−3^ Hz. After this, it is possible to define a dimensionless angular frequency ω* = *ω*_0_/*ω*. This variable ω* is equal to 1 for the quasi-steady non-pulsed case, while it becomes smaller than 1 if the angular frequency is higher, or equivalently if the period is smaller. In Figure 2a, the angular frequency function with different dimensionless angular frequencies is shown. In particular, it is shown that the function with ω* = 1.000 (black line) makes *f*(*ω*,*t*) equal to 0.95 for *t_abl_* = 30 s, then *t_abl_*(ω* = 1.000) = 30 s, remarking again that ω* = 1.000 is taken as the steady-state angular frequency reference case. By reminding that *ω*_0_ = 0.015 rad/s, for the examples presented in Figure 2a, it is shown that *ω* = 0.12 rad/s (light blue, ω* = 0.033) and *ω* = 0.45 rad/s (blue, ω* = 0.125), that roughly correspond to 1.9·10^−2^ Hz and 7.2·10^−2^ Hz, respectively. On the other hand, the lower the dimensionless angular frequency ω*, the smaller is the period, thus the heating frequency of the tissue becomes higher.

After introducing the pulsed functions, procedures to compare pulsed with non-pulsed cases are reported in the following. The parameter used for the sake of comparison is the input voltage *V_in_* since it is the input variable for RF cardiac ablation, and it is easier to manipulate than other variables, for instance, the overall energy. In particular, we assume that in the whole process the product between applied voltage and ablation time is always the same, even if the impedance is not constant due to temperature variations (see Equation (10)). This approach is far less costly of constraining the overall energy; it requires some iterative procedure to appreciate which would be the delivered energy to be constrained for non-pulsating and pulsating cases. Furthermore, as often happens for RF cardiac ablation [8,50,51,52,53,54], it is assumed here that a temperature threshold criterion is used to establish the ablated region, so comparisons between solutions will be done here depending on the temperatures here reached. More details about this criterion will be explained in the Section 3. Despite this, total energy has been checked with energy balances done on the ablated tissue, with values ranging from 28 to 57 kJ referred to all the computations here done. At equal baseline input voltage *V*_0_ and inlet velocity, it has been shown that energy differences are no more than 30% when non-pulsating and pulsating cases are compared. In Figure 2b, the ratio *V_in_*/*V*_0_ is presented as a function of time for both quasi-steady case and ω* = 0.033. If we want that time-average applied voltage ${\bar{V}}_{0}$ in the whole process to be the same, then the areas in Figure 2b have to be equal:

(14) $$\int_{0}^{t_{abl}}{\bar{V}}_{0}t_{abl}\left(\omega^{*}=1\right)\mathrm{dt}=\int_{0}^{t_{abl}}{\bar{V}}_{0}\left[\frac{1}{2}+\frac{1}{2}\cos\left(\omega^{*}t\right)\right]\mathrm{dt}$$

Equation (14) has to be solved with respect to *t_abl_* in order to guarantee that the areas in Figure 2b are the same, thus the following equation can be written:

(15) $$t_{abl}\left(\omega^{*}=1\right)=\frac{t_{abl}}{2}+\frac{\sin(\omega^{*}t_{abl})}{2\omega }=\frac{1}{2}\left[t_{abl}+\frac{\sin(\omega^{*}t_{abl})}{\omega^{*}}\right]$$

By solving this equation for *t_abl_*, one can derive the total time needed for the pulsating process. The solution for this transcendental equation has been obtained in MATLAB and is shown in Figure 2c. Obviously, for ω* = 1.000, *t_abl_* = 30 s, as represented by the dotted line at the bottom of the figure. It is important to observe that for very small dimensionless angular frequencies (say, smaller than about 0.20), ablation time *t_abl_* is roughly equal to two times the ablation time obtained for ω* = 1.000, with an error of less than 10%, since the sinusoidal term of Equation (15) approaches to zero. This means that we can assume for ω* < 0.200 that the ablation time *t_abl_* is two times the ablation time for ω* = 1.000, say *t_abl_*(ω* < 0.200) ≈ 2*t_abl_*(ω* *=* 1.000).

### 2.4. Numerical Modeling and Validation

Governing equations with appropriate boundary conditions and closing coefficients introduced in the previous subsections are solved with the finite-element commercial code COMSOL Multiphysics. Streamline and crosswind diffusion stabilization is used to stabilize the flow field solution, while second-order Lagragian polynomial have been used everywhere to discretize equations. In the simulations, a boundary-layer grid with tetrahedral elements has been used. A number of about 100,000 tetrahedral elements is used in the computations. This number of elements has been checked on temperature profiles evolution vs. time in various points of both catheter and myocardium. Simulations with higher number of elements (say, 150,000 and 200,000) showed that deviations from the used grid are lower than 0.5 °C along temperature vs. time profile. Discretization of time variable has been done with a 0.10 s time step, where convergence has been checked with simulations with 0.05 s and 0.01 s time steps, showing negligible differences on temperature profiles. Finally, equations are solved with an RMS convergence criterion of 10^−5^. Validation of the code has been already presented in Iasiello et al. [11]. In that work, a good agreement was shown with experimental data from Gonzalez-Suarez and Berjano [8].

## 3. Results and Discussion

**Figure 3:**
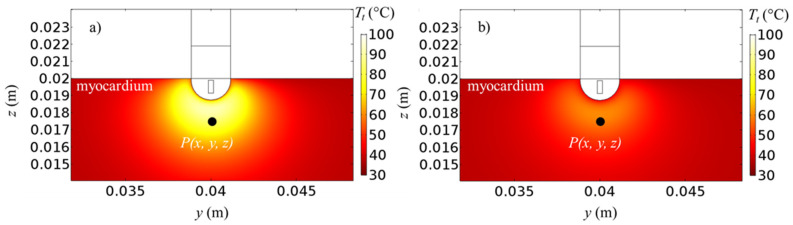
Tissue temperature fields for |**u**|_in_ = 0.05 m/s, *V*_0_ = 22.5 V, (**a**) ω* = 1.000 (*t* = 30 s) and (**b**) ω* = 0.033 (*t* = 60 s).

**Figure 4:**
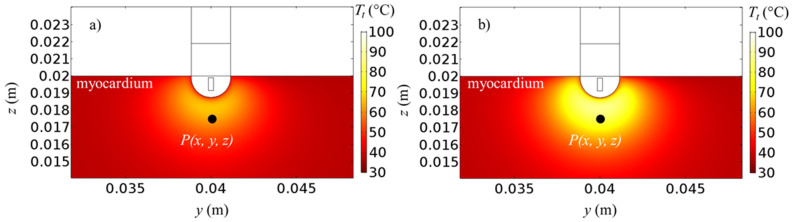
Tissue temperature fields at *t* = 60 s for |**u**|_in_ = 0.05 m/s, ω* = 0.125, (**a**) *V*_0_ = 20 V and (**b**) *V*_0_ = 25 V.

Tissue temperature fields for ω* = 1.000 and 0.033 at *t* = 30 s and 60 s, for *V*_0_ = 22.5 V and |**u**|_in_ = 0.05 m/s are presented in Figure 3. The volume-average symbol < > is dropped for simplicity from now. Higher temperatures are achieved around the catheter, where the heat starts to diffuse. Since the ablation temperature is about 50 °C [8,50,51,52,53,54], all the points in the tissue above about 50 °C are considered to be ablated [8,50,51,52,53,54]. By comparing different heating protocols (Figure 3a,b), it can be seen that the pulsed case provides generally a lower temperature, but the ablation zone remains mostly unchanged since the propagation zone is almost unaffected. This means that similar ablation zones can be achieved by avoiding temperature peaks, as it will be shown later by monitoring temperature evolution for the general point *P*(*x*, *y*, *z*). The effects of the applied voltage on the temperature fields are presented in Figure 4, for |**u**|_in_ = 0.05 m/s, ω* = 0.0125 at *t* = 60 s for *V*_0_ = 20 V and *V*_0_ = 25 V. It can be seen that temperatures are generally slightly higher if a higher voltage is applied. In particular, temperatures of about 100 °C are reached in the catheter proximity for *V*_0_ = 25 V.

**Figure 5:**
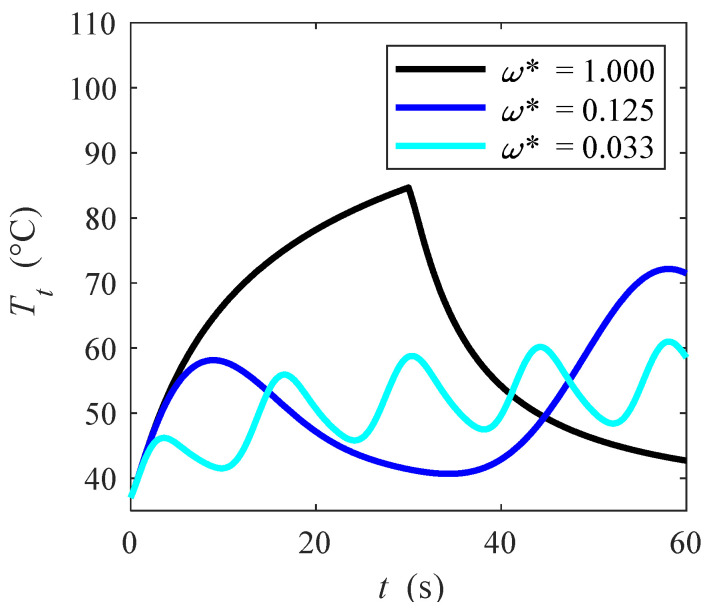
Tissue temperature vs. time for different dimensionless period ω* in P(0.0000, −0.0400, 0.0175).

In order to demonstrate that similar ablation zones can be achieved without any peaks, the variation of the temperature with time at a given point *P*(*x*, *y*, *z*) with *x* = 0.0000 m *y* = −0.0400 m and *z* = 0.0175 m, is shown in Figure 5 for different dimensionless periods ω*, |**u**|_in_ = 0.05 m/s and *V_0_* = 22.5 V. Ablation time *t_abl_* that describe simulations for different ω* has been already introduced in sub Section 2.3 via Equation (13). It is seen that for ω* = 1.000, which corresponds to the quasi-steady state case, ablation temperatures are rapidly achieved in half of the time. However, by varying ω*, it is shown that in some cases, ablation temperature is still reached if pulsed function dimensionless frequency is varied, thus the imposed heat is modulated. In other words, lower temperature peaks are achieved if dimensionless angular frequency is lower, or equivalently frequency is higher. This means that ablation is guaranteed in that point by avoiding any temperature peaks. With references to various dimensionless angular frequencies, one can observe that temperature profiles are almost in phase with applied voltage introduced in Figure 2a. At the beginning of the transient, temperature always increases with time because of the applied voltage, even if this decreases with time (see Figure 2a). After a certain point, even if more heat is supplied, the temperature starts dropping because the blood flow in the blood flow region presents a heat sink effect on the myocardium, thus temperature starts to drop. After reaching a local minimum that is at a time slightly higher than the time at which voltage locally becomes zero, the temperature starts to increase again because the increased voltage takes over blood flow heat sink effect. This effect is periodic through time and occurs for all the dimensionless angular frequency cases here shown.

**Figure 6:**
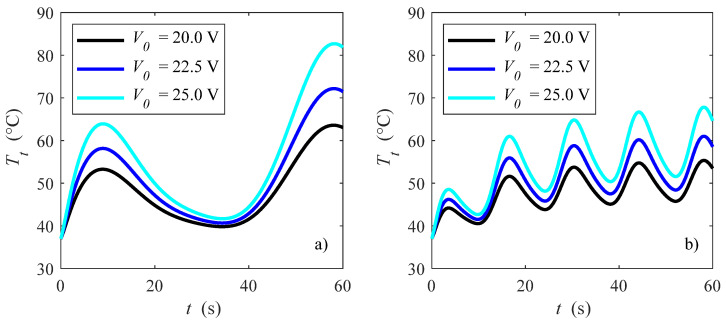
Tissue temperature vs. time for different applied voltages: (**a**) ω* = 0.125 and (**b**) ω* = 0.033 in *P*(0.0000, −0.0400, 0.0175).

Effects of different pulsed dimensionless angular frequencies and applied voltages on temperature evolution at point *P*(0.0000, −0.0400, 0.0175) are presented in Figure 6, with |**u**|_in_ = 0.05 m/s, for ω* = 0.125 and 0.033. For each case, it is shown that higher voltage causes higher temperatures, which might be just a little bit above the ablation temperature mentioned before [8,50,51,52,53,54] with ω* = 0.033 and *V*_0_ = 20 V. By comparing Figure 6a,b, i.e., different angular frequencies ω*, it is shown that higher ω* allows higher temperatures, while lower ω* results in a lower temperature. In particular, temperatures for ω* = 0.125 might reach about 85 °C for the point *P* which is analyzed here. It is very important to module the heat by guaranteeing the same ablation time for the desired zone but without the undesired temperature peaks.

**Figure 7:**
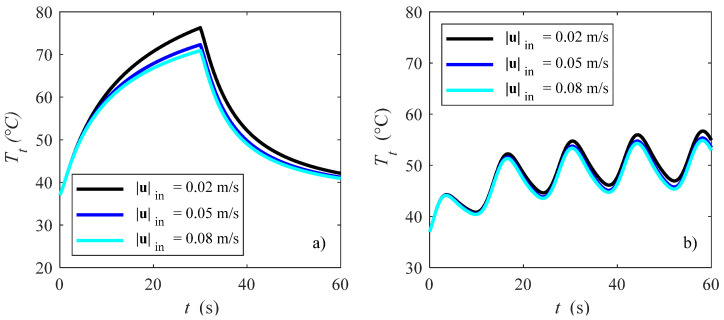
Tissue temperature vs. time for different blood velocities: (**a**) ω* = 1.000 and (**b**) ω* = 0.033 in *P*(0.0000, −0.0400, 0.0175).

In Figure 7, effects of the blood velocity on the temperature fields at the point *P*(0.0000, −0.0400, 0.0175) is shown for *V*_0_ = 20 V and a) ω* = 1.000 and b) ω* = 0.033. With reference to velocity effects, the lower the velocity, the higher the temperatures. This is because for the same applied voltage, i.e., delivered heat power, if the mass flow rate is smaller then the temperature becomes higher due to the smaller ability of the blood to remove heat from the myocardium. Temperatures achieved are much higher for ω* = 1.000 (Figure 7a), reaching about 80 °C at the end of the ablation procedure (*t* = 30 s). If the heat is modulated (Figure 7b), then it is possible to obtain temperatures that are higher than the ablation temperature [54] by avoiding temperature peaks. This means that a modulating heat procedure might improve RF cardiac ablation procedures since ablation zones are similar if one considers the temperature threshold as the damage criterion, as often happens for RF cardiac ablation [8,50,51,52,53,54]. Indeed, these authors employed a 50 °C threshold, and usually a range between 50–56 °C can be reasonably assumed to be the isotherms regions in which ablation is performed for myocardium [8]. Therefore, temperature peaks that could cause tissue damage, roll-off and so on, could be avoided with a pulsating protocol.

Finally, it is also remarked that the present outcomes might be really relevant in clinical practice to avoid any undesired peaks by guaranteeing at the same time a satisfactory result from the ablation. When there is no pulsating procedure—say, ω* = 1.000—very high temperatures are achieved, thus both roll-off and too much large ablation zones might occur. Therefore, if the heat is modulated, one could then still obtain ablation since temperatures are higher than 50 °C—which is a threshold temperature [55]—for reasonable times. The proposed procedure could be also optimized by looking for the best voltage to be applied to have the largest ablation zone without involving any healthy tissue. This optimization could involve also other factors to be accounted for in the model, say for instance hypertension, hyperthyroidism [56], lifestyle factors [57], alcohol consumption, smoking [58], and channelopathies [59,60]. Additionally, within these particular conditions, the present model could be adapted to account for particular circumstances; for instance, hypertension can be accounted for by assuming different pressure drops through the investigated domain. Another aspect that is relevant in clinical applications is about the connection of the present procedure with the well-established zero X-ray ablation approach [61]. This approach comes from the fact that during ablation one would have some non-negligible stochastic and deterministic effects on health due to radiation exposure [62,63]. Therefore, if one modulates applied heating, then voltage, the overall induced electromagnetic fields can be damped.

## 4. Conclusions

Effects of modulating heat for RF cardiac ablation are analyzed in this work. The electrical problem is solved by employing the Laplace equation for radiofrequencies. Mass, momentum, and energy equations are described for both the blood region and myocardium, while the heat equation is used for the whole antenna. A porous media LTNE model has been used to characterize the flow and temperature fields in the myocardium. Thermophysical properties, together with porous media properties, have been taken from the pertinent literature and temperature dependence of properties has also been taken into account. Modulating heat effects are considered by employing a cosine function in which angular frequency is varied, at equal delivered energies. Governing equations were solved with a finite-element commercial code. Results are presented in terms of the temperature field for different applied voltages, blood flow velocities, and angular frequency ratios. It has been shown that higher voltages and lower blood velocities guarantee higher temperatures. With reference to the modulated heat cases, different angular frequencies have been analyzed. It has been shown that the modulated heat could reduce temperature peaks by still guaranteeing the desired ablation zone.

## Figures and Tables

**Table 1:** Thermophysical properties and porous media characteristics employed in this work.

Element (Material)	*σ* (S/m)	*k* (W/m K)	*ρc* (MJ/m^3^ K)	*K ·* 10^11^ (m^2^)	*ε*	*h_c_* (W/m^2^ K)	*S_v_* (1/mm)
Electrode (Pt-Ir)	4.6∙10^6^	71	2.838				
Thermistor (Glass Fiber)	1.0∙10^−5^	0.038	0.027				
Catheter (Polyurethane)	1.0∙10^−5^	0.026	0.073				
Cardiac chamber (Blood)	0.667	0.541	4.180				
Myocardium (Tissue/Blood)	Equation (10)/ 0.667	Equation (11)/ 0.541	Equation (12)	5.6	0.10	170	50

## Data Availability

Data of the present research are available upon request to the authors.

## Nomenclature

*C_H2O_*	water content
*C*	heat capacity (J kg^−1^ K^−1^)
**E**	electric field (V m^−1^)
*h_c_*	interfacial heat transfer coefficient (W m^−2^ K^−1^)
*k*	thermal conductivity (W m^−1^ K^−1^)
*K*	permeability (m^−2^)
*p*	pressure (Pa)
$\dot{Q}$	heat rate (W)
${\dot{Q}}_{RF}$	radiofrequency heat generation (W m^−3^)
*S_v_*	specific surface area (m^−1^)
*t*	time (s)
*T*	temperature (K)
**u**	velocity vector (m s^−1^)
*V*	voltage (V)
*x, y, z*	cartesian coordinates (m)
**Greek letters**	
*ε*	porosity
*λ*	water latent heat of vaporization (J kg^−1^)
*μ*	viscosity (Pa s)
*ρ*	density (kg m^−3^)
*σ*	electric conductivity (S m^−1^)
*ω*	angular frequency (rad s^−1^)
**Subscripts**	
0	reference
*abl*	ablation
*b*	blood
*c*	catheter
*g*	gas
*eff*	effective
*ev*	evaporation
*in*	inlet
*l*	liquid
*t*	tissue
